# Holistic health record for Hidradenitis suppurativa patients

**DOI:** 10.1038/s41598-022-11910-5

**Published:** 2022-05-19

**Authors:** Paola Maura Tricarico, Chiara Moltrasio, Anton Gradišek, Angelo V Marzano, Vincent Flacher, Wacym Boufenghour, Esther von Stebut, Matthias Schmuth, Wolfram Jaschke, Matjaž Gams, Michele Boniotto, Sergio Crovella

**Affiliations:** 1 Department of Advanced Diagnostics, Institute for Maternal and Child Health - IRCCS Burlo Garofolo, Trieste, Italy; 2 Dermatology Unit, Fondazione IRCCS Ca’ Granda Ospedale Maggiore Policlinico, Milan, Italy; 3 Department of Medical Surgical and Health Sciences, University of Trieste, Trieste, Italy; 4 Department of Intelligent System, Jožef Stefan Institute, Jamova Cesta 39, 1000 Ljubljana, Slovenia; 5 Department of Pathophysiology and Transplantation, Università Degli Studi Di Milano, Milan, Italy; 6 Laboratory CNRS I2CT/UPR3572 Immunology, Immunopathology and Therapeutic Chemistry, Strasbourg Drug Discovery and Development Institute (IMS), Institut de Biologie Moléculaire Et Cellulaire, University of Strasbourg, Strasbourg, France; 7 Department of Dermatology, University of Cologne, Kerpenerstr. 62, 50935 Cologne, Germany; 8 Department of Dermatology, Venereology and Allergy, Medical University of Innsbruck, Anichstrasse 35, Innsbruck, Austria; 9 INSERM, IMRB, Translational Neuropsychiatry, F-94010, University Paris Est Créteil, Créteil, France; 10 Biological Sciences Program, Department of Biological and Environmental Sciences, College of Arts and Sciences, University of Qatar, Doha, Qatar

**Keywords:** Genetics, Immunology, Molecular biology, Stem cells, Diseases, Health care, Medical research, Pathogenesis

## Abstract

Hidradenitis suppurativa (HS) is a recurrent inflammatory skin disease with a complex etiopathogenesis whose treatment poses a challenge in the clinical practice. Here, we present a novel integrated pipeline produced by the European consortium BATMAN (Biomolecular Analysis for Tailored Medicine in Acne iNversa) aimed at investigating the molecular pathways involved in HS by developing new diagnosis algorithms and building cellular models to pave the way for personalized treatments. The objectives of our european Consortium are the following: (1) identify genetic variants and alterations in biological pathways associated with HS susceptibility, severity and response to treatment; (2) design in vitro two-dimensional epithelial cell and tri-dimensional skin models to unravel the HS molecular mechanisms; and (3) produce holistic health records HHR to complement medical observations by developing a smartphone application to monitor patients remotely. Dermatologists, geneticists, immunologists, molecular cell biologists, and computer science experts constitute the BATMAN consortium. Using a highly integrated approach, the BATMAN international team will identify novel biomarkers for HS diagnosis and generate new biological and technological tools to be used by the clinical community to assess HS severity, choose the most suitable therapy and follow the outcome.

## Introduction

Hidradenitis suppurativa (HS), also known as acne inversa, is a chronic, inflammatory, recurrent, debilitating skin disease. With a prevalence in Europe of 0.8 (varying between 0.5% and 1.3%) and a diagnosis often underestimated and usually delayed up to 7.2 ± 8.7 years, HS can’t be considered as a rare disease^1^ [https://www.orpha.net/consor/cgi-bin/OC_Exp.php?lng=en&Expert=387 Women are more frequently affected than men (female:male ratio, 3:1) and have more likely genitofemoral lesions^2^. The disease onset occurs usually during adolescence or early adulthood, resulting in frustration, embarrassment and depression. Very often, the severe forms start manifesting in early adolescence/puberty thus resulting in several inflammatory lesions over the years. Thus, early diagnosis and adequate, stage-adapted therapy is essential to avoid a destructive course of disease. Clinically, HS is characterized by inflammatory nodules that progress into abscesses and draining tunnels with foul smelling^3^.

HS is usually a sporadic disease, but may occur as a familial disorder^4^. A familial form is reported in 40% of cases showing an autosomal dominant mode of inheritance with incomplete penetrance in some cases^2,5^. In a minority of patients, HS can be associated with other immune-mediated inflammatory diseases or inherited conditions, presenting as “syndromic” HS^6^. The main autoinflammatory syndromes characterized by the presence of HS are pyoderma gangrenosum (PG), acne and suppurative hidradenitis (PASH), pyogenic arthritis, PG, acne and suppurative hidradenitis (PAPASH), psoriatic arthritis, PG, acne and suppurative hidradenitis (PsAPASH), pustular psoriasis, arthritis, PG, synovitis, acne and suppurative hidradenitis (PsAPSASH) and PG, acne, suppurative hidradenitis, and ankylosing spondylitis (PASS)^7^.

Cigarette smoking and obesity are crucial trigger factors in HS and both have been directly correlated with the severity of this disease. Although clinically follicular hyperkeratosis and bacterial superinfection are common features, the molecular pathogenesis of HS remains to be clarified. Therefore, HS treatment is somehow empirical and includes topical (antibiotics, keratolytics, antiseptics), intralesional (corticosteroids), surgical (deroofing, wide local excision), and systemic (antibiotics, retinoids, biologics, immunosuppressants, metformin, antiandrogens) interventions. In particular, biologics, i.e. antibodies directed against TNF-alpha, IL-17, IL-23, IL-1, and latest generation immunosuppressants, i.e. JAK-inhibitors, are promising options awaiting a better pathogenetic rationale and a P4 medicine approach (predictive, preventative, personalized, participatory) to be introduced in the routine clinical practice.

Taking into account the current gap of knowledge in HS etio-pathogenesis, patients’ efficient and timely diagnosis as well as tailored clinical follow-up, the purpose of this manuscript is to present a novel integrated pipeline produced by the ERAPerMed European consortium BATMAN (Biomolecular Analysis for Tailored Medicine in Acne iNversa) aimed at increasing the knowledge in HS etio-pathogenesis with the objective of translating the findings in the tailored clinical follow-up of HS patients.

The BATMAN consortium has been constituted joining the efforts of dermatologists with three active clinical Units from Milano (Italy), Innsbruck (Austria) and Cologne (Germany), geneticists (Trieste, Italy), immunologists and molecular cell biologists (Paris and Strasbourg, France) with the pivotal participation of informatics technology (IT) partners (Ljubljana, Slovenia).

In detail, this European Consortium aims at:

1. identifying genetic variants and alterations in biological pathways associated with HS susceptibility, severity and response to treatment;
2. designing in vitro two-dimensional epithelial cell and tri-dimensional skin models to unravel the HS molecular mechanisms;
3. producing holistic health records (HHR) to complement medical observations by developing a smartphone application to remotely monitor the physical and psychological wellbeing of patients and advise them on physical activity and dietary and smoking habits.

Here we will describe the main activities developed by different members of the Consortium including the IT approach that, integrating medical records, genetic results, immunology and cell biology findings will contribute to providing a tailored diagnosis for HS patients as well as paving the way for personalized treatments, thus starting to respond to the main patients’ needs and changing the patients’ care strategies used in clinical practice.

## Aim 1: Identify genetic variants and alterations in biological pathways associated with HS susceptibility, severity and response to treatment

Genetic diagnosis of diseases requires a physical examination, personal medical history records, family health history and laboratory tests, including genetic testing. The occurrence of the same condition among family members is the key factor to be considered: pedigree analysis of the families with more than one member affected is the key clue for determining the patterns of disease inheritance.

As mentioned above, HS familial cases are reported in 40% of cases, more than one-third of patients. Whether HS manifests as an inherited disease and what is the frequency depends upon several factors: difficult and often delayed diagnosis of HS, caused by the lack of knowledge of this disease, absence of personal and family health history investigation, incomplete penetrance of this disease and also the unwillingness of other family members to participate in the study^5^.

Genetic mutations in the γ-secretase enzyme subunits (*NCSTN*, *PSEN1*, *PSENEN*) have been reported in HS familial cases suggesting that HS could be considered a monogenic disease with autosomal dominant inheritance pattern and incomplete penetrance. Mutations in these genes lead to an impairment of the Notch pathway and/or of inflammasome signaling^8,9^.

Whole exome sequencing (WES) of 11 HS families has been performed by P. Theut Riis et al.^10^ The authors reported several variants of uncertain significance that segregated with the disease within these families. These findings suggest that familial HS can be regarded as a polygenic autoinflammatory condition and, only in a minority of cases, as a monogenic disease^10^. Despite this, further studies and functional validations are necessary to highlight the genetic landscape of familial HS.

In a cross-sectional study in a Dutch twin cohort, Van Straalen et al. observed an high heritability of HS (77%) and also environmental factors as significant contributors to the susceptibility of this disease, supporting a multifactorial etiology of this skin disorder^11^.

In sporadic and syndromic HS cases, the contribution of genetic factors is still an active research area, and several genetic/functional studies are ongoing to unravel the pathogenesis of HS and its syndromic forms. A range of genetic changes have been associated with HS pathogenesis, including variations in genes involved in autoinflammation, vitamin D metabolism and keratinization pathway^12^.

The Table 1 shows all the genes involved in HS known to date.

**Table 1.** Summary of the genes involved in HS.

Gene	Encoding protein	Disease	References
*APH1B*	Aph-1 homolog B	Familial HS	^10^
*DEFB103*	Defensin Beta 3 (hBD3)	Sporadic HS	^13^
*DEFB4*	Defensin Beta 2 (hBD2)	Sporadic HS	^13^
*FGFR2*	Fibroblast Growth Factor Receptor	Sporadic HS + Nevus comedonicus and Syndromic HS	^4,14^
*GJB2*	Gap Junction Protein Beta 2, Connexin-26	Sporadic HS + Keratitis-ichthyosis-deafness syndrome and Syndromic HS	^14, 15^
*IL-12Rb1*	Interleukin-12 Receptor Subunit Beta-1	Sporadic HS	^16^
*LPIN2*	Lipin 2	Sporadic HS	^12^
*MEFV*	MEFV innate immunity regulator	Familial HS and Syndromic HS	^4, 12, 17^
*MYD88*	Myeloid Differentiation Primary Response Protein MyD88	Sporadic HS	^18^
*NCSTN*	Nicastrin	Familial HS, Syndromic HS and HS + Dowling-Degos disease	^4, 15, 19, 20^
*NLRC4*	NLR Family CARD Domain Containing 4	Syndromic HS	^15^
*NLRP12*	NLR Family Pyrin Domain Containing 12	Syndromic HS	^12^
*NLRP3*	NLR Family Pyrin Domain Containing 3	Syndromic HS	^12^
*NOD2*	Nucleotide Binding Oligomerization Domain Containing 2	Familial HS and Syndromic HS	^12, 15, 17, 19^
*OCRL1*	Inositol polyphosphate 5-phosphatase	Sporadic HS + Dent disease 2	^21^
*OTULIN*	OTU Deubiquitinase With Linear Linkage Specificity	Syndromic HS	^15^
*POFUT1*	Protein O-Fucosyltransferase 1	Syndromic HS and HS + Dowling-Degos disease	^4, 19, 22^
*PSEN1*	Presenilin 1	Familial HS	^9, 23^
*PSENEN*	Presenilin Enhancer Protein 2	Familial HS, Syndromic HS and HS + Dowling-Degos disease	^9, 19, 24, 25^
*PSTPIP1*	Proline-Serine-Threonine Phosphatase Interacting Protein 1	Syndromic HS	^15, 19, 26, 27^
*TNF*	Tumor Necrosis Factor	Sporadic HS	^28^
*WDR1*	WD Repeat Domain 1	Syndromic HS	^15^

So, it’s a widely accepted fact that genetics plays a role in the susceptibility to develop HS and to modulate the clinical phenotype. Moreover, patients’ genetic background can have an impact in pharmacogenetics/pharmacogenomics; indeed, this kind of research is important to understand the genetic influence on the organism response to different drugs, different doses, and the timing of drugs’ elimination.

Liu et al. performed GWAS, designed for HS response to adalimumab (anti-TNFa), in 445 HS patients; the authors identified a single-nucleotide polymorphism (SNP) (rs59532114) in the intron (chr18:63162799, GRCh38.p13) of the *BCL2* gene, associated with adalimumab response^29^. This SNP minor A allele is associated with increased *BCL2* gene expression and its augmented protein level in the hair follicle, thus suggesting a potential role of apoptosis’ regulation in the pathophysiology of adalimumab response in HS patients.

Previously, the same authors observed that HLA-DRB1*03 and HLA-DRB1*011 allele conferred an increased risk for developing anti-adalimumab antibodies in HS patients, while HLA-DQB1*05, HLA-DRB1*01 and HLA-DRB1*07 allele might protect against anti-adalimumab antibodies formation^30^. The formation of anti-drug antibodies to adalimumab can lead to its therapeutic ineffectiveness and these findings can lead the switch to other, more effective therapies.

So, genetically guided decisions of tailored therapies aim to enhance treatment success rate with a consequent improvement of patients’ quality of life.

The discovery of differentially expressed genes (DEGs) that induce alterations in biological molecular pathways is highly necessary for improving understanding of HS pathogenesis and also of HS treatment. Coates M. et al. observed a dysregulation of antimicrobial peptides between lesional and unaffected skin of HS patients, analyzing HS skin transcriptomes from previously published studies. This observation allowed to confirm the key role of the innate immunity in the HS pathogenesis and also to pave the way for development of new therapies based on supplementation/activation of antimicrobial peptides^31^. On the other hand, Mariottoni et al. identified an upregulation of genes involved in interferon and antimicrobial defense signaling. They also observed monocyte/macrophage dysregulation, with single cell RNA sequencing (scRNA-seq) analysis of skin tissue; according to the authors this observation could pave the way for the identification of potential novel therapeutic targets^32^.

Considering all these findings, HS has no clear and well-defined mode of inheritance, due to the polygenic and multifactorial nature of the disease. Thus, the following question arises: how far are we from the identification of genetic variants useful for disease’s mechanistic understanding as well as for personalized diagnosis and treatment? Could the integration of data between genetic variants and DEGs help to understand HS pathogenesis? How genetics can help in sporadic and syndromic cases?

In the attempt of contributing to unraveling these questions, the dermatologists of the BATMAN consortium, collected and accurately clinically phenotyped HS familial and sporadic cases, as well as syndromic HS. Saliva is collected from all HS patients for DNA extraction in order to perform genetic analyses (single nucleotide polymorphisms arrays and/or whole exome sequencing); instead, skin punch biopsies are collected only from HS familial and syndromic cases in order to identify DEGs and perform histological analyses.

## Aim 1: Methods

### Patients recruitment

The inclusion criteria for patient enrollment are the compliance to the diagnostic criteria for HS. Three criteria must be fulfilled:

1.Typical lesions: deep-seated painful nodules, abscesses, bridged scars and draining fistulae as well as open, tombstone double-ended comedones (pseudocomedones). Usually, multiple lesions are present that facilitate the diagnosis. It is, however, important to avoid misdiagnosis with nondiagnostic elements, such as simple folliculitis.
Predilection sites: axillae, inframammary and/or intermammary folds, groin, perineal region, or buttocks. Lesions may appear ectopically (i.e. face, trunk, ears) but they must involve the areas for which the disease has a predilection to meet the diagnosis.
History of chronicity and recurrence: temporary lesions initially recur in the predilection areas only to turn chronic later in the course of the disease. Two recurrences over a period of six months have been used as a useful diagnostic criterion.

All three criteria must be present for HS diagnosis, and considering the recurrence/chronicity, an observation period may be necessary before the definitive diagnosis^33^.

Based on clinical observations, six phenotypes have been proposed: (1) regular, (2) frictional furunculoid, (3) scarring folliculitis, (4) conglobata, (5) ectopic and (6) syndromic^34^. However, Dudink et al. suggested that the ectopic and syndromic types do not have specific clinical features and could be categorized as one of the other phenotypes^35^. Finally, Frew et al. presented a revised phenotype classification system: (1) typical (previously regular subtype) and (2) atypical that includes scarring folliculitis and conglobata subtypes^36^. However, given the clinical heterogeneity of HS, further sub-classifications under the “atypical” need additional investigations. Syndromic disease has the potential to become a third classification. Patients with HS syndromic are characterized by concomitant manifestations, such as PG and arthritis; however, other clinical consensus are required to define the symptomatology sufficient for a syndromic phenotype diagnosis. It remains unclear whether a syndromic phenotype should remain restricted to PASH and PAPASH syndromes or if other autoimmune/autoinflammatory syndromes such as Familial Mediterranean Fever and Dowling Degos Disease should also be included^25,36,37^.

Interestingly, familial HS cases show a severe disease phenotype not entirely matching with the one typical of “sporadic” HS, as mentioned above^38^.

Accurate disease severity assessment and classification of HS patients are mandatory for guiding therapeutic decisions as well as for patient stratification. Zouboulis et al. have established IHS4 (International Hidradenitis Suppurativa Severity Score System), a dynamic HS score^39^. This IHS4 score (points) = (number of nodules multiplied by 1) + (number of abscesses multiplied by 2) + [number of draining tunnels (fistulae/sinuses) multiplied by 4]. A score of 3 or less signifies mild HS, a score of 4–10 corresponds to moderate HS and a score of 11 or higher reveals severe HS.

This proposed score can be used complementary and simultaneously with HiSCR (Hidradenitis suppurativa Clinical Response) score and both can be calculated easily in daily clinical practice and clinical trial settings.25 The HiSCR is defined as a ≥ 50% reduction in inflammatory lesion count (sum of abscesses and inflammatory nodules) and no increase in abscesses or draining fistulas in HS when compared with baseline. This score was only recently created but it is a promising clinical endpoint to evaluate therapeutic outcomes in patients with Hidradenitis suppurativa.

### Sample collection

All biological samples are obtained, in all clinical centers, with written informed consent and institutional review board approval, in agreement with the Helsinki Declaration and local legislations (the study was approved by the institutional review boards of “Comitato Etico Unico Regionale of Friuli Venezia Giulia, Institute for Maternal and Child Health IRCCS Burlo Garofolo” Italy, of “Comitato etico Milano Area 2 IRCCS CA' GRANDA the Policlinico Maggiore Hospital” Italy, of “Medical University of Innsbruck” Austria and of “Medical University of Cologne” Germany).

**Figure 1:**
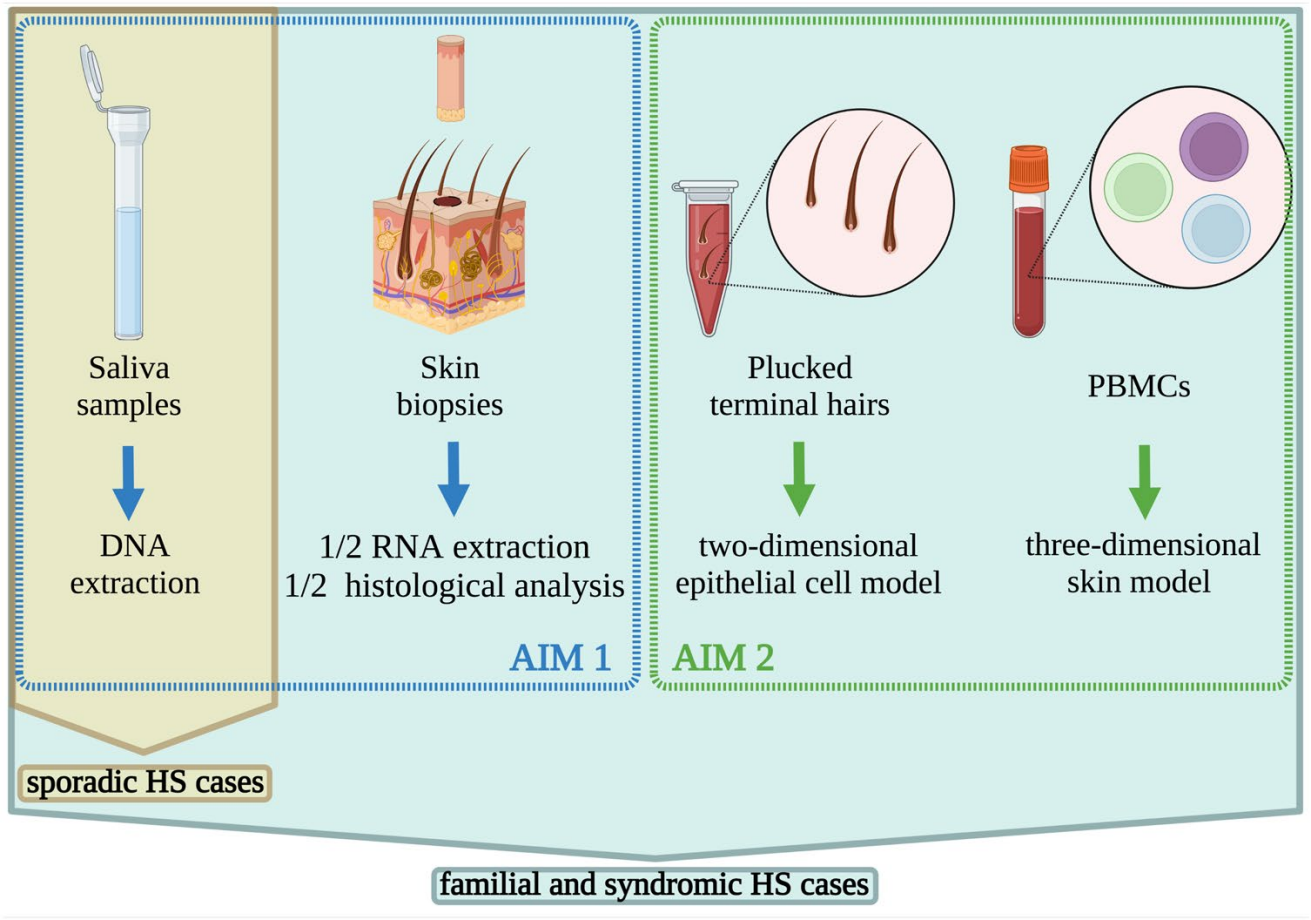
Schematic representation of samples collection. From familial and syndromic HS cases are collected: saliva for DNA extraction (AIM1), skin punch biopsies (AIM1) half for RNA extraction and other half for histological analysis, plucked terminal hairs (AIM2) for two-dimensional epithelial cell model, and blood cells (AIM2) for three-dimensional skin model; from sporadic HS cases saliva is collected.

In detail, from familial and syndromic HS cases the following samples are collected: saliva (for DNA extraction), skin punch biopsies (half for RNA extraction and other half for histological analysis), plucked terminal hairs (for two-dimensional epithelial cell model, aim 2) and blood cells (for three-dimensional skin model, aim 2); from sporadic HS cases saliva (for DNA extraction) is collected (Fig. 1).

Saliva is collected using the Oragene-DNA (Ottawa, Canada) kit, following the manufacturer’s protocols, and stored at room temperature.

Skin biopsies are taken from lesional regions of HS patients. One half of the biopsy is placed directly in RNAlater (Qiagen, Hilden, Germany) and stored at − 20 °C; the other half of the biopsy is embedded in paraffin and maintained at room temperature.

Plucked terminal hairs in anagen phase are placed immediately in DMEM low calcium (Gibco, Thermo Fisher Scientific) with 1X gentamicin/amphotericin (Gibco, Thermo Fisher Scientific). Hairs can be stored at room temperature and can be processed up to three days after isolation.

B and T cell populations are analyzed by flow cytometry from fresh blood samples. Peripheral blood mononuclear cells (PBMCs) are stored in nitrogen and are used either as a source of monocytes (see 3D skin models) or in functional assessment of T cell responses.

### Genetic analyses

DNA is extracted from saliva using the Oragene-DNA (Ottawa, Canada) kit following the manufacturer’s protocols.

Single nucleotide polymorphisms (SNPs) arrays, using the Illumina Infinium® Global Screening Array-24 v1.0 Kit, is performed on all recruited HS patients in order to identify:

1. Disease-shared genomic regions, comparing patients with healthy controls;
2. Disease severity, comparing patients stratified based on Hurley score;
3. Novel loci associated with successful response to anti-TNF treatment, comparing HS responders with non-responders based on HiSCR.

Moreover, all HS syndromic cases and selected HS familial cases, the most distant familiar HS cases (i.e. proband vs. cousin or vs. nephew), are further investigated by Whole Exome Sequencing (WES) searching for novel candidate genes/mutations. WES with 100X of expected coverage is performed in outsourcing by Macrogen (Seoul, Korea).

### Transcriptome analyses

RNA is extracted from one half of skin biopsy with the RNeasy Lipid Tissue kit (Qiagen, Hilden, Germany) using TissueLyser II (Qiagen, Hilden, Germany), following the manufacturer’s protocols. Transcriptome analysis is performed with RNA sequencing (RNAseq), in outsourcing by Macrogen (Seoul, Korea), in order to identify DEGs and alterations in biological molecular pathways, to confirm the genetic variations identified with WES.

### Genomic and transcriptomics data integration

Single-omics datasets, such as genomic and transcriptomic, could fail to fully untangle the complexity of a disease, so HS can be better understood by integrating the multi-omics datasets. In detail, omics data integration will allow a broad characterization of the molecular mechanisms, an identification of key molecular pathways involved in HS susceptibility, onset, clinical course and severity. For this purpose, we have developed the PlatOMICs, a platform capable of automatically deciphering the information derived from the public repositories as well as from the scientific literature interpreting and integrating with the findings obtained by omics analysis in HS patients^40^.

### Skin histology and integrated OMICs data validation

The other half of skin biopsy is used to perform conventional histological analysis as well immunohistochemistry aimed at validating the genetic and transcriptomic data and to identify any variation in expression and localization of particular proteins.

## Aim 2: Design in vitro two-dimensional epithelial cell and tri-dimensional skin models to unveil the molecular mechanisms occurring in HS pathogenesis

### Two-dimensional epithelial cell model

Recently, apocrine sweat gland involvement in HS has been discarded. Neither differences in apocrine sweat gland size and density or in its morphology have been observed in HS patients. Infundibular plugging of the folliculosebaceous apocrine apparatus, on the other hand, has been observed in the early phase of the disease^41,42^.

More recently, Dunstan et al. observed hyperplasia of the infundibular ORS, early “tendril” formation, and keratin plugging with infundibular dilatation in punch biopsies and excisional skins from 65 HS patients^43^. Interestingly, in fully developed lesions, cells expressing outer root sheath (ORS) markers, such as KRT17, 19 and nestin are capable of proliferating and infiltrating the dermis then differentiating in corneocyte (expressing KRT10), and form “cysts” filled with antigenic keratin. Dunstan et al. also identified KRT15, 19 and nestin positive cells in the tendrils thus showing that ORS cells are capable of an uncontrolled proliferation and invasiveness becoming corneocytes (KRT10+) or going back to the multipotency. This finding was corroborated by scRNA-seq performed earlier by Marohn et al.^44^ The results from the scRNA-seq analysis revealed that cells forming tendrils shared common transcripts with the cells of infundibulum/sweat duct, therefore expressing high levels of genes specific for sweat and sebaceous glands, albeit their morphology and differentiation program resembled interfollicular keratinocytes.

Loss of hair follicle stem cells identity is a peculiar characteristic of HS, and it seems to be independent to the disease's varied inflammatory response that may be initiated by chemokines and cytokines expressed by cells in the tendrils and fueled by keratin spilled from the final cysts^43,44^.

These findings imply that ORS cells could be the main culprit in HS. Two-dimensional ORS cultures have been already used to show an increased expression of inflammatory cytokines and chemokines in HS patients and decreased expression of the constitutive antimicrobial peptide beta defensin-1 (hBD-1)^45^. Transcriptome analysis of ORS cells, isolated from 6 HS patients and cultured in a defined medium, showed a differential expression of genes involved in cell proliferation and differentiation, and an upregulation of the DNA damage response and cell cycle G2/M checkpoint pathways in HS^46^.

The main question here addressed is: how far can we go in personalizing HS patients’ follow-up by using their biological material (i.e. ORS cells)? What are the advantages of designing personalized treatment based on cells from patients? What are the limitations (if any) or advantages related to ORS differentiation for each patient?

ORS cells present a non-invasive and autologous source of stem cells. Thus a model aiming to unveil the HS molecular mechanisms leading to the disorder should incorporate these cells as main actors. Isolating cells from a patient suffering from HS and comparing their properties with cells from a healthy control may be useful to characterize HS genetics (both at genome and transcriptome levels) and protein profiles. This could provide a solution in identifying new elements involved in this skin pathologic condition.

Two-dimensional collection and culture of ORS from HS patients have some major advantages. First, ORS bring us one step closer to a model organism to study the disease, and they are relatively easy to manipulate. Second, in contrast to blood cells and skin fibroblast, the latter requiring invasive extraction methods, ORS represent an easily accessible cell type thus providing an opportunity for patients to collect and send their own hair samples to the laboratory. This is important for HS patients who are eager to participate in HS research, but fell off from clinical follow-up as they were discouraged for absence of improvement in their condition. Third, ORS is very resourceful in terms of in vitro cultivation. It harbours a heterogeneous cell pool, including stem cells mainly present in the bulge area^47^. ORS can be isolated from hair follicles even after 5 days from hair plucking, allowing the time for transporting the cells from the clinical center to the laboratory just sending the cells conserved in appropriate medium by express courier. Remarkably, during the pandemics the possibility for the clinicians to send the ORS to the specialized laboratory by courier, allowed the continuity of the research.

Finally, ORS cells isolation doesn't need a long enzymatic digestion, commonly applied in skin samples in order to loosen the tissue around the follicles prior to dissection, which may damage the cells.

Some drawbacks in using ORS cells technique may also be observed. This technique is time consuming and an expensive method for cell isolation and amplification. In addition, primary cells may reach senescence and differentiation after 5–8 passages, which restrains its utilization for precision medicine.

In the BATMAN project ORS cells isolated from patients are being used to unveil the HS molecular mechanisms and to identify biological pathways affected by the disease thus helping in identifying novel genes associated with disease susceptibility. ORS cells present a non-invasive and endless autologous source of stem cells. Isolating cells from a patient suffering from HS and comparing their properties with cells from a healthy control may be useful to characterize HS genetic and protein profiles. This could present a solution in identifying new elements involved in the skin pathologic condition. Since HS phenotypes vary between patients, ORS cells should be ideally isolated from each patient willing to participate in the project. However, for this project we have chosen to collect hairs from familial and syndromic HS patients for whom WES has already been performed to identify and/or confirm a candidate genetic variant associated with the disease.

RNA sequencing of low passage ORS cells cultured in defined media can serve to identify DEGs that will be clustered in pathways and assembled with genetic findings by using AI tools.

Finally, it should be said that nowadays the ORS study approach is for research use only, and still far from being applied in the routine patients’ follow-up due to the limitations mentioned above.

### Three-dimensional skin models

Human models that accurately reproduce HS in vitro would represent a substantial asset for investigations on physiopathology, drug screening and personalized medicine approaches^48^. Ex vivo culture of patients’ skin explants replicates known characteristics of the disease, and incubation with different HS-targeting drugs yielded responses that were consistent with clinical trial results^49,50^. Lesional skin may be obtained upon surgical excision, which remains frequent for HS patients. Nevertheless, such samples are difficult to maintain in sterile culture conditions because they are highly septic. Collecting non-lesional skin biopsies may pose ethical issues if they are of a sufficient size to allow ex vivo assays. Therefore, there is a need for alternative models that could be grown from a limited amount of patients' cells.

**Figure 2:**
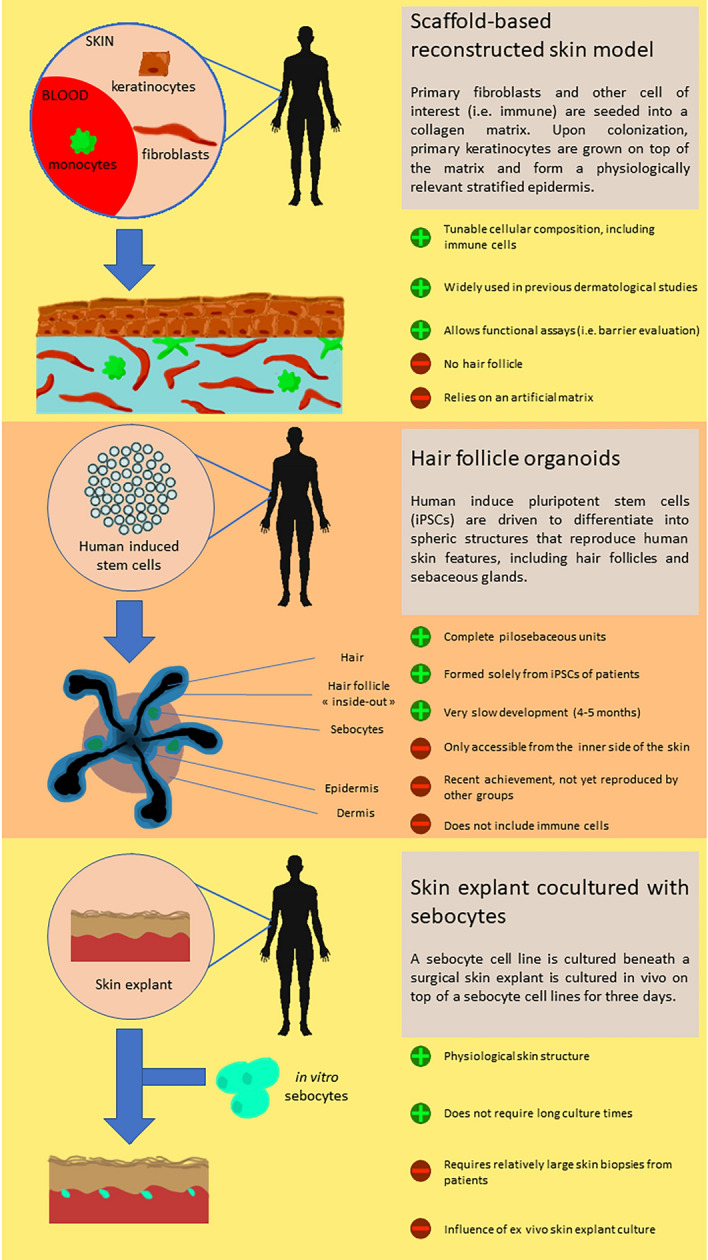
Representation of the three types of 3D skin models relevant for HS studies. The scaffold-based reconstructed skin model, the hair follicle organoids and the skin explant cocultured with sebocytes.

Reconstructed skin models have evolved from tissue engineering methods, which initially aimed at reproducing a functional organ in vitro with the goal of transplanting it to compensate for a deficiency. For the skin, it started from the production of epidermal grafts for patients suffering extensive burns wounds. More recently, protocols using genetically modified keratinocytes have been implemented to correct genetic insufficiencies that dramatically alter the skin barrier function^51^. In parallel, similar culture techniques have been applied to generate in vitro models, and these tissue reconstructions are now often employed in research and in biosafety evaluations. Here, we will present different forms of 3D skin models (Fig. 2) and discuss the relevant features to resolve poorly understood aspects of HS pathogenesis.

As an interface organ, the organization of the skin is composed to create a physical barrier protecting against harmful substances and pathogens. The ultrastructure of the epidermal compartment can be convincingly reproduced by keratinocytes alone, which spontaneously stratify when cultured at the air–liquid interface. Commercially available healthy epidermis models (i.e. Episkin) can be challenged by chemical substances. This technique also led to building models from keratinocytes of atopic dermatitis patients^52^. In the context of HS, hair follicle rupture and the subsequent dermal invasion by proinflammatory molecules and microorganisms might result from impairment of the barrier function. Even in the absence of a hair follicle, this fragility or increased permeability could be advantageously tested in an epidermal model constructed from HS patients with genetic defects suspected to alter the cohesion between keratinocytes. Moreover, gamma-secretase deficiencies associated with HS cases are expected to impact keratinocyte growth and might lead to aberrant epidermal formations when cultured in vitro. Complementing this approach by CRISPR/Cas9 genetic engineering would be an important tool to assess the effect of genetic alterations introduced in healthy cells. Conversely, if abnormal epidermal structure is observed with patient cells bearing a polymorphism of interest, CRISPR/Cas9 could be used to correct the mutation and determine how important it was in the anomaly.

By including a dermal compartment based on an artificial matrix, full-thickness skin models have an extended potential to reproduce more complex functions, i.e. innervation, vascularization or immunocompetence. In particular, immune barrier function of the skin requires a dense network of mononuclear phagocytes, which prevent pathogen invasion either by direct elimination (innate immunity) or by enabling the effector function of T and B cells (adaptive immunity). These sentinels comprise Langerhans cells (LCs) in the epidermis and different subsets of dendritic cells (DCs) and macrophages in the dermis^53^. To reproduce the key function of immunocompetence, several skin models integrate DCs, LCs or macrophages differentiated from hematopoietic stem cells of umbilical cord blood or peripheral blood monocytes^54,55^. Indeed, at least part of HS pathogenesis might be due to the patient’s exacerbated potential to perpetuate inflammation in the skin, following a microbial challenge. This could be conveniently investigated in an immunocompetent 3D model, in which immune cells would be in a physiologically relevant position to interact with their neighbours, possibly leading to a self-fueling inflammation. The outcome of such experiments could be compared with the signature of surgically removed HS lesions by multiparameter analysis, i.e. single-cell transcriptomics. Unfortunately, neutrophils, which typically infiltrate HS lesions, remain very difficult to maintain ex vivo and therefore cannot be introduced in a skin model. Yet, one could expect to detect a strong expression of chemokines that attract neutrophils in a relevant HS skin model. Finally, the cutaneous environment (cellular contacts, soluble factors, microbiome) shapes the capacity of DCs to direct adaptive immune responses. In a skin model including monocyte-derived DCs from HS patients, we could evaluate their potential to bias T-cell differentiation towards Th17-dominated responses typical of this disease.

Despite constant efforts to ameliorate their complexity and physiological relevance, tissue-engineered skin models retain a major shortcoming so far: the absence of skin appendages. Notably, hair follicles are believed to play a central role in HS pathogenesis. They are located at the dermo-epidermal interface, constitute a dense epidermal niche, and their cyclic renewal must be tightly regulated to avoid structural dysfunction such as occlusion or rupture. Recently, a novel culture method has been able to produce skin organoids harbouring structurally relevant hair follicles^56^. This was done in the absence of a solid culture scaffold and relied on the self-organization capacity of precursor cells. Although constructing this model entails a long and complex procedure and still represents a challenge, yet this important innovation is likely to be included as a tool to study hair follicle-based diseases in the near future. Finally, these organoids expand from a limited number of easily accessible hair follicle stem cells which differentiate into a large variety of cell types. Therefore, enzymatically dissociated hair follicle organoids may represent a reliable source of keratinocytes and fibroblasts, which are obtained from invasive biopsies.

In the context of HS, another crucial part of the pilosebaceous unit is the sebaceous gland. Anomalies in this appendage are believed to play in follicular obstruction and dysbiosis. The influence of sebaceous glands can be mimicked in vitro by coculturing healthy skin explants with a sebocyte cell line^57^. This model has been recently applied to HS skin reconstruction, using patient cells^58^. Interestingly, the company Labskin Creations (Lyon, France) proposes a tissue-engineered model integrating hIPSC-derived sebocytes, which assemble as structurally relevant sebaceous glands in the dermal compartment (https://www.labskincreations.com/SeboSkin.aspx).

## Aim 2: Methods

### In vitro two-dimensional epithelial cell model

**Figure 3:**
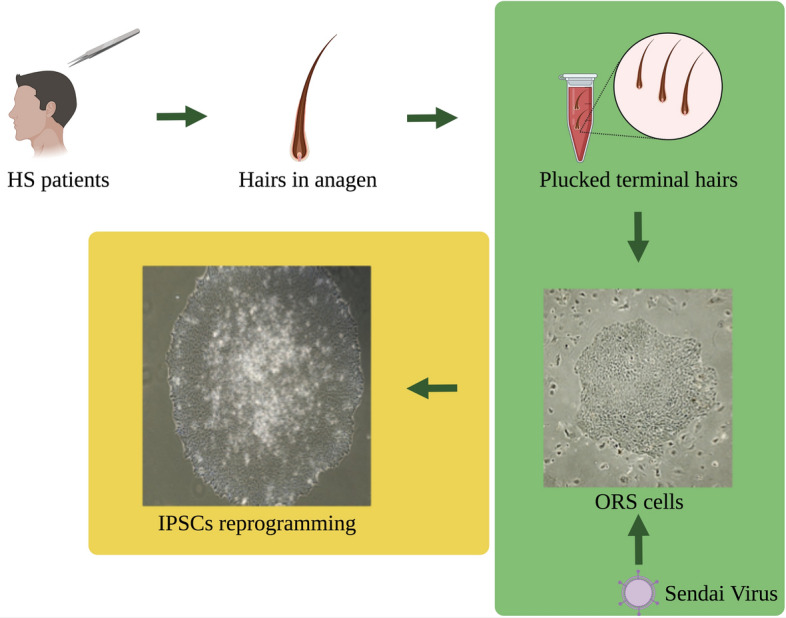
Schematic representation of hairs collection, ORS isolation and IPSCs reprogramming. Pluked termial hairs in anagen phase are collected from familial and syndromic HS cases. ORS cells isolated from plucker hair are aplificated and reprogrammated using Sendai virus to obtain Induced Pluripotent Stem Cells (iPSCs).

ORS cells isolated from plucked hairs are amplified and maintained following the protocol described by Rheinwald and Green in presence of feeder cells 3T3-J2 (kindly donated by Dr. Y. Barrandon) (Fig. 3)^59^. Cells are further expanded in a defined medium and we have had consistent results with EpilifeTM (ThermoFisher Scientific,Villebon-sur-Yvette, France), CNT-07 (CELLnTEC, Bern, Switzerland) and DermaCult™ Keratinocyte Expansion Medium (StemCell Technologies, Saint Égrève, France). After amplification cells are stored at − 150 °C for further use.

Cells with novel candidate mutations or with a mutation in genes already associated with HS are used to obtain Induced Pluripotent Stem Cells (iPSCs) (Fig. 3). Cells are reprogrammed using the CytoTune iPS 2.1 Sendai Reprogramming kit (ThermoFisher Scientific) with two different ratios of Sendai virus particles containing Yamanaka factors. Six to seven days after infection, cells are plated in 100-mm culture plates treated with gelatin and pre-seeded with irradiated Mouse Embryonic Fibroblasts (MEFs). iPSCs colonies are picked manually, subcloned, cultured and characterized following published protocols. Karyotyping of iPSC clones is performed by SNPs-microarray analysis and genetic mutation confirmed by Sanger sequencing.

### Three-dimensional full-thickness skin model

iPSCs derived from ORS of HS patients are used to differentiate keratinocytes and fibroblasts. The cells incorporated in control models are obtained from healthy skin donors or from iPSCs with CRISPR/Cas9-corrected genetic variants. Innate immune sentinels, i.e. macrophages, dendritic cells and Langerhans cells, are derived in vitro from monocytes of healthy donors or HS patients. Fibroblasts and immune cells are sequentially introduced into a collagen-chitosan matrix, mimicking the dermal compartment. Then, keratinocytes are seeded on top of the matrix and allowed to differentiate into an epidermis upon exposure to air–liquid interface. The resulting models are analyzed by immunohistochemistry, ELISA and flow cytometry.

## Aim 3: Produce holistic health records (HHR) to complement medical observations by developing a smartphone application

The use of smartphone applications related to health has expanded substantially over the past decade, with smartphones being daily companions for a majority of the population. While most commonly-known health-related applications focus on aspects such as exercise (“fitness trackers”) or nutrition, independent of involvement of a medical doctor, there is also a segment of application that has found its use in the clinical practice. In the field of dermatology, a recent review grouped the applications according to their functions as teledermatology, self-surveillance/diagnosis, disease guide, and general dermatology reference^59^. Considering the teledermatology applications, one approach uses a live consultation with a dermatologist, while the other one requires the patient to take a photo with the smartphone and send it to a server for a dermatologist to review later. Some of these applications are supported by national or other insurance plans^60^.

Self-surveillance/diagnosis apps often harness the processing power of a smartphone in combination to its plethora of sensors. In dermatology, the most useful sensor is clearly the phone camera. Image recognition algorithms have been employed to detect and monitor skin cancer, and the applications run either automatically or in association with a dermatologist^61^.

There is ongoing research on algorithms that are able to distinguish between different skin diseases. Pangti et al. developed a deep-learning method that was able to distinguish between 40 common conditions^62^. The study included hidradenitis suppurativa, where the algorithm achieved about 80% sensitivity and almost a 100% specificity in a cross-validation experiment.

The advantage of the self-surveillance/diagnosis approaches is their ability to provide the patient the information fast, without the need to visit a dermatologist. However, this is also a major shortcoming, since the algorithms have various degrees of accuracy in detection or classification, and furthermore can be biased based on the dataset that was used to train them. Coupled with the abundance of misinformation and unvetted applications, this calls for a very cautious handling of such applications, which should ideally only be used together with a professional medical counsel. As patients are typically not equipped to critically assess and evaluate the information found online, the doctors should work with them to educate them and to inform them where to find appropriate resources^60^.

There are several online resources that provide dermatological information. For example, the Dermatology Atlas (https://play.google.com/store/apps/details?id=com.andromo.dev706301.app782141) and VisualDx (https://apps.apple.com/us/app/visualdx/id348177521) are applications that contain extensive collections of photographs of different conditions and are useful resources for both medical students and practicing doctors. Resources for patients are also available in different forms and typically also in the patient’s language. An example is the Italian platform https://lapellesicura.it that offers information about several dermatological conditions, including HS.

In the BATMAN project, a smartphone application (https://play.google.com/store/apps/details?id=si.ijs.batman&hl=en) and a web-based platform (https://batman-project.eu/) for research purposes were used, to collect patient data, in order to build a holistic picture of patients with Hidradenitis suppurativa.

**Figure 4:**
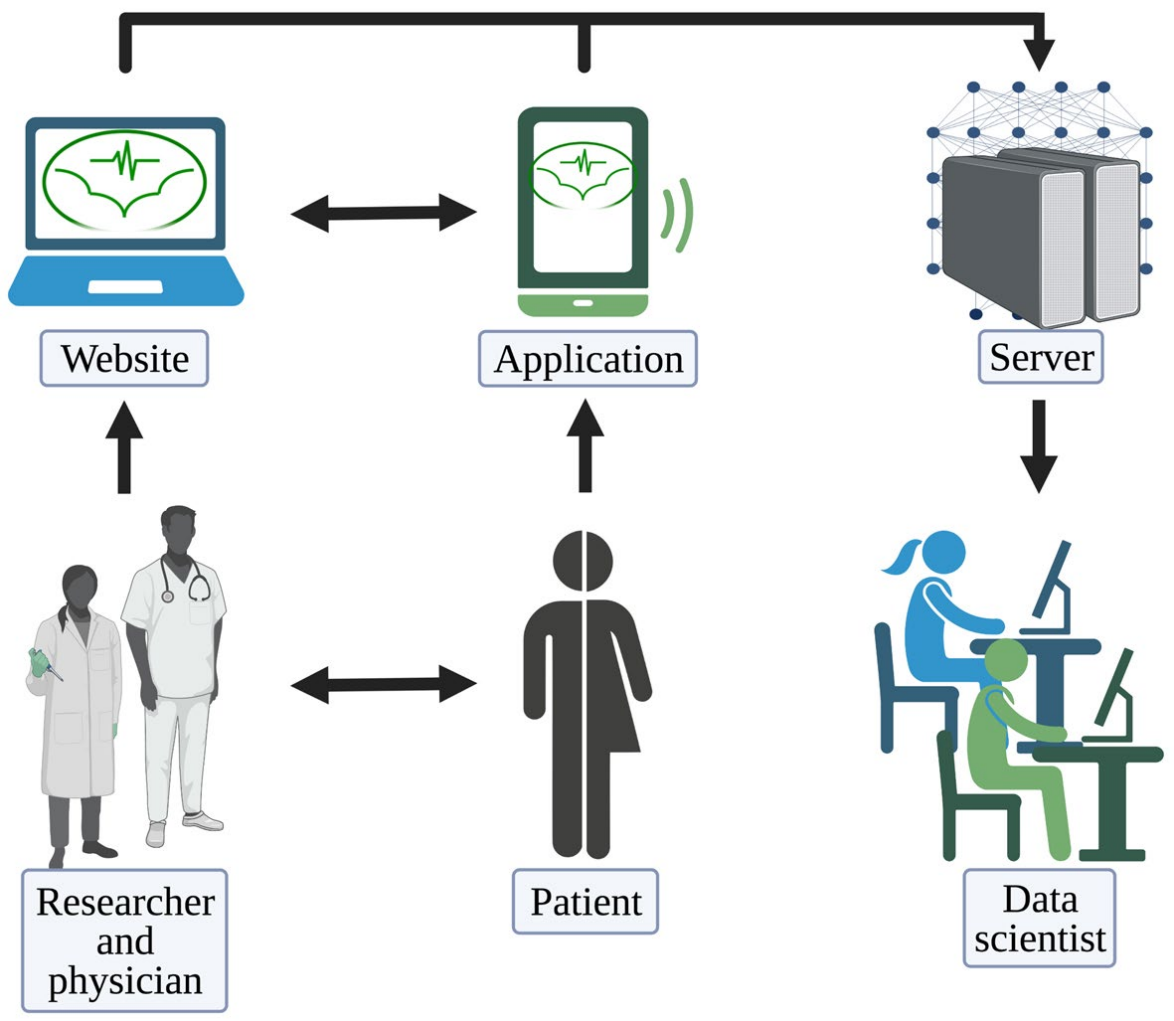
Schematic representation of AIM3. The interactions beetween physicians, patients and data scientists with the platform.

It has been previously demonstrated that it is beneficial to look at the HS patients from different viewpoints: medical, functional genetic, and lifestyle. All three to some degree influence the occurrence and the severity of the medical condition, therefore it is beneficial for a doctor to have a look at the complete data, e.g. holistic data. For this purpose, we developed a web platform for doctors and patients, coupled to a smartphone application for the patients that allows us to obtain all three types of data (Fig. 4).

## Aim 3: Methods

Medical data is obtained in the form of a standard electronic health record (EHR) and is uploaded into the platform by the physician, using a unique patient identifier. Genetic data, likewise, comes in a standard format and is uploaded using the same identifier.

Lifestyle data is collected in real time from the patients through a dedicated smartphone application, and is of two types. Self-reported data is obtained using questionnaires that the patient fills in when prompted. In the food preferences questionnaire, the patients report their dietary preferences, namely how often they eat certain types of food. This form is filled in by the patient only once in the study as it is expected that the preferences are unlikely to change during the duration of the pilot. Additional questions, that are asked only once, related to the alcohol consumption and smoking habits, whether they like to exercise in a company, and the daily hours of sleep. On the other hand, there are two questionnaires that the patient may be asked by the doctor to fill in on several occasions, to track the development of the medical condition. These are the Dermatological Quality of Life (DQoL) questionnaire and the Major Depression Inventory (MDI).

In addition to the self-reported data, the smartphone application records physical activity of the patient, which is done using a step counter plug-in. Physical activity is on one hand important as a way to cope with the HS and on the other hand, it can be used as a proxy by the doctor to see how severe the medical condition that the patient is currently experiencing is. Namely, as HS involves the lesions developing in the intertriginous body areas, pain is one of the most important problems; the patient will feel pain while moving, thus will likely rest more. Conversely, an increase in a patient's movement on a daily basis likely corresponds to a decrease in pain intensity and an improvement in HS and in the quality of life.

The communication between the smartphone and the platform takes place through a secure protocol. The doctor issues the patient a unique username and the password and the application only sends the answers to the questionnaires and aggregated daily activity data, ensuring that no additional information that could be used to identify the patient from that data (such as the IPs or location data) is shared with the platform. Since the study is carried out in the EU, a special care was paid to assure that all steps are GDPR-compliant^63^. The data used for analysis is thus anonymized, stripped of any possible personal identifiers—the medical doctor is the only person who knows the identity of their patients.

Future work could include adding supplementary information to the HHR. In studies of support groups on Facebook, Lombardo et al.^64,65^ took advantage of the users’ posts and comments to extract information about emotional states and social interactions. They also studied the correlations of emotional states with the time of the day or season of the year. Such automated emotion recognition is beneficial as it reduces the need for questionnaires, although the implementation of such type of patient monitoring is complicated due to privacy concerns. On the other hand, monitoring of stress using a wristband has already been explored^66^. A wristband as an activity-monitoring device has further advantages when compared to a smartphone, as the user can wear it all the time, even at night, and can record physiological data such as heart rate and sweating. The choice of the smartphone as patients’ device to collect data, instead of the wristband is related to the need of using the most available tool (the vast majority of people do possess a smartphone); a wristband could be then provided to HS patients in the future, this wristband will be also linked to the smartphone to allow real-time communication between patients and the physicians.

## Conclusions

As examples of BATMAN bench to patients’ application of genetic analysis, based on integrated WES, we have been able to identify the biologic pathways associated with syndromic HS in patients from the IRCCS Ca’ Granda Ospedale Maggiore Policlinico of Milan^67^. We identified genetic variants impairing the Vitamin D (calciferol) metabolism in HS patients^68^. Based on our findings the dermatologists started to administer Vitamin D to the patients, with a significant improvement of the skin conditions, thus ameliorating their quality of life. We also developed the PlatOMICs, a novel IT- based in house tool for OMICs integrated analysis, basically genomics and transcriptomics, in the context of skin diseases^40^; our PlatOMICs platform allows users, to integrate and re-analyze OMICs information, looking more in deep the molecular actors playing a role in the pathogenesis of HS and its syndromic forms. By analyzing the WES of 10 Unrelated Patients with Syndromic HS, we further concluded that syndromic HS can be considered as a polygenic autoinflammatory condition^15^.

In addition to all of this, we must consider that the platform is built in a way that it allows for future extensions. For example, monitoring the daily activity of the patient can give the doctor direct feedback on how effective the current treatment is, and the DQoL and MDI questionnaires provide direct insight into the patient's mood. Using an in-app messaging function, the doctor can then send the patient’s advice.

The BATMAN project is an European project with impact on the national health systems; our consortium is seeking to prompt interventions aimed at supporting HS patients, with particular attention to women and adolescents, promoting not only clinical or diagnostic actions but also support (i.e. psychological) for a better inclusion of HS patients in the social context, thus ameliorating their quality of life.

This project aims at providing early diagnosis and personalized clinical follow-up for HS patients by identifying novel biomarkers. We surmise that through genetic profiling and continuous monitoring of well-being using a dedicated application and platform, we can also propose novel stratification methods that clinicians can use to assess HS severity, choose the therapy and follow the outcome. Research on in vitro two-dimensional cell and tri-dimensional skin models support preclinical findings by validating genetic variants’ role in HS and by generating novel models to understand pathophysiology allowing the exploration of different therapeutic approaches.

## Data Availability

Supporting data is available at SRA: https://www.ncbi.nlm.nih.gov/sra/PRJNA801118.
